# Schiff-Linked PEGylated Doxorubicin Prodrug Forming pH-Responsive Nanoparticles With High Drug Loading and Effective Anticancer Therapy

**DOI:** 10.3389/fonc.2021.656717

**Published:** 2021-03-25

**Authors:** Jian Song, Bingbing Xu, Hui Yao, Xiaofang Lu, Yang Tan, Bingyang Wang, Xing Wang, Zheng Yang

**Affiliations:** ^1^ Department of Pathology, The Seventh Affiliated Hospital of Sun Yat-Sen University, Shenzhen, China; ^2^ Department of Sports Medicine, Peking University Third Hospital, Institute of Sports Medicine of Peking University, Beijing, China; ^3^ Eye Hospital, China Academy of Chinese Medical Sciences, Beijing, China; ^4^ Beijing National Laboratory for Molecular Sciences, Institute of Chemistry, Chinese Academy of Sciences, Beijing, China; ^5^ University of Chinese Academy of Sciences, Beijing, China

**Keywords:** pH-responsive nanoparticles, prodrug, Schiff base, control release, drug delivery

## Abstract

Developing efficacious drug delivery systems for targeted cancer chemotherapy remains a major challenge. Here we demonstrated a kind of pH-responsive PEGylated doxorubicin (DOX) prodrug *via* the effective esterification and Schiff base reactions, which could self-assemble into the biodegradable micelles in aqueous solutions. Owing to low pH values inside the tumor cells, these PEG-Schiff-DOX nanoparticles exhibited high drug loading ability and pH-responsive drug release behavior within the tumor cells or tissues upon changes in physical and chemical environments, but they displayed good stability at physiological conditions for a long period. CCK-8 assay showed that these PEGylated DOX prodrugs had a similar cytotoxicity to the MCF-7 tumor cells as the free DOX drug. Moreover, this kind of nanoparticle could also encapsulate small DOX drugs with high drug loading, sufficient drug release and enhanced therapeutic effects toward MCF-7 cells, which will be benefited for developing more drug carriers with desirable functions for clinical anticancer therapy.

## Introduction

Biodegradable nanoparticles have been intensively utilized as promising candidates for targeted cancer therapy over a long period, because they possess the increased drug solubility, prolonged circulation time, improved pharmacokinetic properties, good bioavailability, enhanced tumor accumulation at the tumor sites, high therapeutic effects and low systemic side effects (1–8). Among these various nanomedicines such as nanoparticles, polymeric micelles, vesicles, nanogels, liposomes and polymer conjugates, prodrug-based nanoparticles have drawn much more attention due to their simple structure, feasible preparation and great potential in clinical translation (9–11).

Doxorubicin (DOX) is a most widely used anticancer drug approved by FDA, which has been applied to the treatment of many neoplasms, but its severe toxic side effects, low bioavailability and high doses requirement greatly limit its clinical use (12, 13). Therefore, significant progress has been made towards the fabrication of ideal DOX delivery system with improved drug utilization at the action sites and reduced side effects in non-target tissues. Although the liposomal formulations of DOX have been approved for clinical use and many polymeric nanoparticles loading DOX molecules are being evaluated in clinical trials (14–18), these DOX-loaded nanocarriers have some inherent drawbacks like low drug loading, premature burst release and inactive carriers to patients, which may ascribe to the high polarity and hydrophilicity of DOX molecules. To overcome this unintended and undesirable leakage, chemically-linked drug carriers offer an alternative for the effective anti-tumor treatment. For example, Wen et al. had prepared a high-density DOX component *via* covalent decoration on the nanocarrier, which could be effectively released in the acidic tumor tissue and significantly improved chemotherapy efficacy (19).

Polyethylene glycol (PEG) is one of the most widely used hydrophilic polymer approved by FDA with negligible toxicity and low immunogenicity, which has been expected to effectively reduce the non-specific uptake by the reticuloendothelial system (RES), prolong circulation time and allow for specific tumor-targeting *via* the enhanced permeability and retention (EPR) effect (20–24). Thus, PEGylated doxorubicin prodrugs have shown distinctly therapeutic advantages including better antitumor activity and fewer side effects than free drugs. However, many reported PEG-DOX prodrugs presented some inherent drawbacks like relatively low drug loading capacity, incomplete drug release and insensitive manipulation (25–27). To ensure the high-effective intracellular drug release, it is important to design and develop more stimuli-responsive prodrugs that can possess precise target ability and controllable drug release inside the target cells upon changes in physical and chemical environments. Wherein, the significance of pH change in the tumor cell environment is recognized as a most popular chemical stimuli to tailor drug release in aqueous solutions. For example, Ding et al. developed a cRGD-decorated pH-responsive polyion complex (PIC) micelle for intracellular targeted delivery of DOX to upregulate tumor inhibition and reduce toxicity (28–33). Although Sui et al. had reported a PEG-DOX prodrug with an acid-labile hydrazone bond for cancer chemotherapy, this drug system displayed a weak pH response with approximately 12% release content at pH 5.0 even for 3 days, which may induce the antitumor efficiency in the first place and lose the opportunity for early treatment (26).

**Figure 1:**
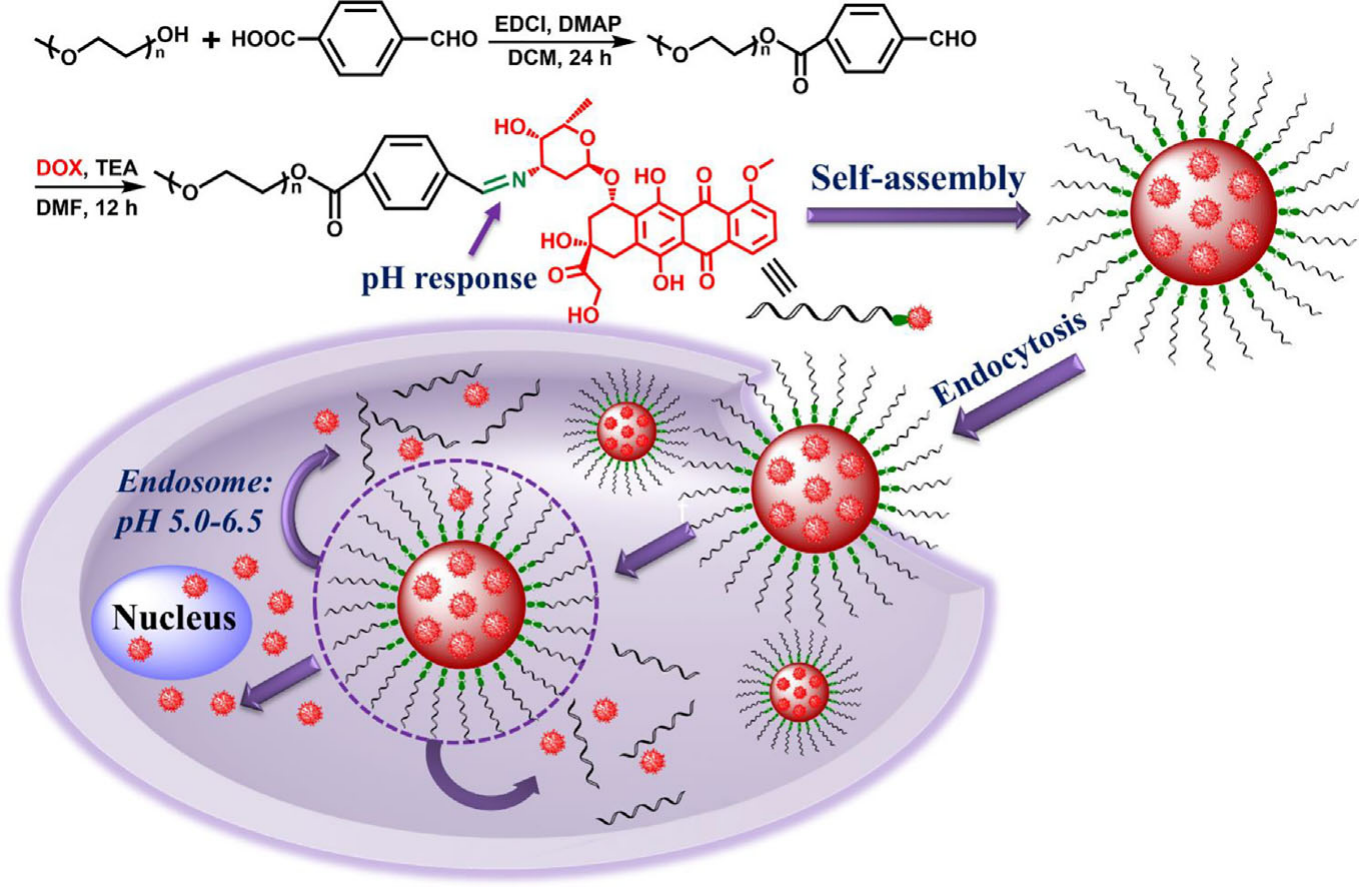
Synthetic route of pH-responsive PEG-Schiff-DOX polymer and activated intracellular drug release from the pH-sensitive self-assembled nanoparticles.

In this study, we designed and prepared an amphiphilic polymer drug conjugate (PEG-Schiff-DOX) *via* an acid-sensitive Schiff base bond, which could self-assemble into pH-responsive nanoparticles in solutions. These PEGylated nanoparticles had a well-defined structure, suitable architecture and size, high and fixed DOX loading content, good biocompatibility, stability and storage (**Figure 1**). The bridged Schiff bases could keep the structural integrity at normal physiological condition but endow the aggregates disassembly in response to faintly acidic environment. CCK-8 assays showed that these PEG-Schiff-DOX nanoparticles demonstrated outstanding antitumor activity compared to free DOX against human breast cancer cells (MCF-7). Additionally, benefitting from the drug carrier with the encapsulation ability, a new DOX-based PEG-Schiff-DOX polymer with programmed DOX release behavior was obtained, which may result in high drug concentration, long treatment period and enhanced therapeutic effect. Thus, we believe that these prodrug nanomedicines will hold promise to an alternative translational DOX formulations for cancer chemotherapy.

## Materials and Methods

### Materials

Poly(ethylene glycol) methyl ether (PEG-OH, M_n_ = 750 g/mol, Alfa Aesor), 4-carboxybenzaldehyde (98%, J&K), 4-(dimethyl-amino)-pyridine (DMAP, 99%, Aldrich), 1-(3-dimethylaminopropyl)-3-ethylcarbodiimide hydrochloride (EDCI, 99%, Energy Chemical), triethylamine (TEA, 99%, Beijing Chemical Works), hydrochloric acid, sodium sulfate, sodium bicarbonate, n-hexane and ether were purchased from Beijing Chemical Works. Doxorubicin·HCl (DOX·HCl) was purchased from Beijing Zhongshuo Pharmaceutical Technology Development Co., Ltd. Dichloromethane (DCM) and dimethylformamide (DMF) were purified by stirring over calcium hydride for 24 h followed by distillation. All other reagents were purchased from Sigma-Aldrich and used as received without further purification. Cells were supplied by China Infrastructure of Cell Line Resource.

### Characterizations

Nuclear magnetic resonance (NMR) spectra were recorded on a Bruker 400 MHz spectrometer with tetramethylsilane (TMS) as the internal reference. Transmission electron microscopy (TEM) images were obtained on a JEM-2200FS microscope (JEOL, Japan). A 5 μl droplet of self-assembled solution was dropped onto a copper grid (300 mesh) coated with a carbon film, followed by drying at room temperature. Dynamic light scattering (DLS) spectra were obtained on a commercial laser light scattering spectrometer (ALV/DLS/SLS-5022F) equipped with a multi-τ digital time correlator (ALV5000) and a cylindrical 22 mW UNIPHASE He-Ne laser (λ_0_ = 632.8 nm) was used. All data were averaged over three time. The laser light scattering cell was held in a thermostat index matching vat filled with purified and dust-free toluene, with the temperature controlled to within 0.1°C. Fluorescence measurement was carried on a Hitachi F4600 photo-luminescent spectrometer with a xenon lamp as a light source. Confocal laser scanning microscopy (CLSM) images were obtained on a Zeiss LSM 510 microscope.

### Synthesis of the PEG-CHO Polymer

PEG-OH (0.75 g, 1 mmol), 4-carboxybenzaldehyde (0.18 g, 1.2 mmol), EDCI (0.24 g, 1.2 mmol) and DMAP (25 mg, 0.2 mmol) were added into a 50 ml round-bottomed flask equipped with a magnetic stirring bar, followed by the addition of 20 ml of freshly dried DCM to fully dissolve all the solids. After the stirring for 24 h at room temperature, the organic phase was collected and washed with 2 M HCl aqueous solution, saturated sodium bicarbonate solution, saturated NaCl aqueous solution and DI water for several times and dried over anhydrous Na_2_SO_4_. The final product was precipitated into ether/hexane for three times to afford the white powder with 90.1% yields.

### Synthesis of the PEG-Schiff-DOX Polymer

PEG-CHO (0.27 g, 0.31 mmol), DOX·HCl (0.15 g, 0.25 mmol) and TEA (70 μl, 0.5 mmol) were dissolved in 10 ml of DMF solution under a nitrogen atmosphere. The mixture was refluxed with vigorous stirring for 12 h. After removing the solvent under the vacuum, the crude products were resolved in DCM and washed with saturated NaCl aqueous solution and DI water for several times and dried over anhydrous Na_2_SO_4_. The final product was precipitated into ether/hexane for three times to afford the dark-red powder with 66.4% yields.

### Formation and Self-Assembly of the Polymeric PEG-Schiff-DOX Nanoparticles

A typical self-assembly solution was prepared as following: PEG-Schiff-DOX (5 mg) was first dissolved in DMF (1 ml), then the deionized water (4 ml) was added dropwise into the solution at the rate of 0.05 ml/min *via* a syringe pump. The colloidal dispersion was further stirred for another 2 h at room temperature during the self-assembling process. The organic solvent was removed by dialysis (MW cutoff, 1 kDa) against deionized water for 3 days. These PEG-Schiff-DOX nanoparticles were obtained and characterized by TEM and DLS measurement.

### Stability and pH-Responsive Degradation of the PEG-Schiff-DOX Nanoparticles

In PBS (pH 7.4) and fetal bovine serum (FBS) solutions, we measured the transformation of size distributions for PEG-Schiff-DOX nanoparticles in order to study the stability and resisting protein adsorption ability. First of all, the self-assembled nanoparticles were dispersed in PBS and 20 wt% of FBS solutions with the concentration of 1 mg/mL, respectively. For maintaining the constant temperature of 37°C, the size distributions were recorded by DLS for each 12 h.

For determining the pH response of PEG-Schiff-DOX nanoparticles, a certain amount of phosphate buffered saline (PBS) with different pH values (5.0 and 7.4) was added into 10 ml of PEG-Schiff-DOX nanoparticles (1 mg/ml) under mildly stirring at 37°C. After 4 h, morphological and dimensional changes of nanoparticles were characterized by TEM and DLS measurement.

### *In vitro* DOX Release From the PEG-Schiff-DOX Nanoparticles

pH-triggered DOX release measurement was conducted as below: dispersed PEG-Schiff-DOX nanoparticles were added into a dialysis membrane tube (MW cutoff, 1 kDa), which was then incubated in 30 ml of PBS (pH 5.0 and 7.4) solutions at 37°C in a shaking water bath. pH-triggered DOX release profiles were determined by measuring the UV-vis absorbance of solutions at 480 nm. All DOX-release experiments were conducted in triplicate and the results were expressed as average data with standard deviations.

### Cellular Uptake and Intracellular Localization of MCF-7 Cells Incubated With PEG-Schiff-DOX Nanoparticles and Free DOX Drug

MCF-7 cells were plated on microscope slides in a 96-well plate (5 × 10^4^ cells/well) using Dulbecco’s Modified Eagle Medium (DMEM) containing 10% FBS. After incubation for 24 h, the cells were incubated with prescribed amounts of free DOX (8 μg/ml) and PEG-Schiff-DOX nanoparticles (equivalent to 8 μg/ml DOX) for 1 and 4 h at 37°C and 5% CO_2_. Then the culture medium was removed and the cells on microscope plates were washed for three times with PBS. After fixing with 4% paraformaldehyde overnight, the MCF-7 cells were observed under a confocal laser scanning microscopy (CLSM) with excitation at 488 nm for DOX.

### CCK-8 Assay

The cytotoxicity of PEG-Schiff-DOX and DOX-encapsulated PEG-Schiff-DOX nanoparticles was studied by CCK-8 assay using MCF-7 cells. Cells were seeded onto a 96-well plate at a density of 1×10^4^ cells per well in 200 μl of DMEM containing 10% FBS and further incubated for 24 h (37°C, 5% CO_2_). The medium was replaced by 90 μl of fresh DMEM medium containing 10% FBS, and then various concentrations (10^−3^–10 μg/ml) of micelle suspensions in PBS (pH 7.4) solutions were added. After incubation for another 24 h and removal of culture media from cell culture plates, 100 μl of fresh culture media and 10 μl of CCK-8 kit solutions were immediately added and homogeneously mixed and then incubated for 4 h in a CO_2_ incubator. Finally, 100 μl of solutions were put into 96-well plate. The optical density of each well at 450 nm was read by a microplate reader. Cells cultured in DMEM medium containing 10% FBS (without exposure to nanoparticles) were used as controls.

## Results and Discussion

### Synthesis and Characterization of PEG-Schiff-DOX Prodrugs

**Figure 2:**
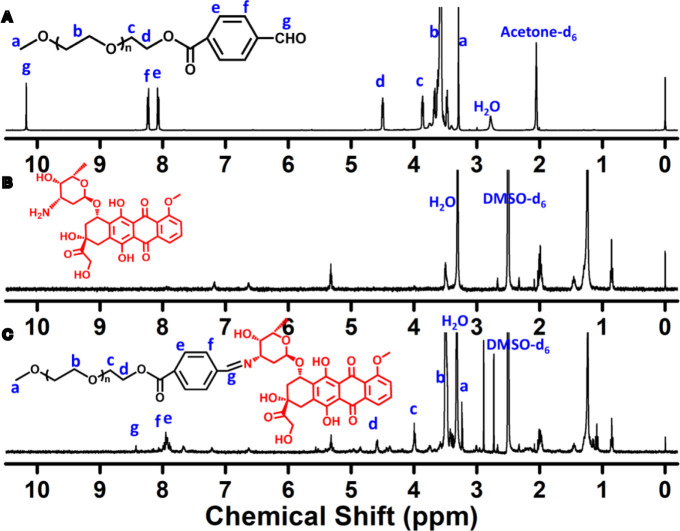
^1^H NMR spectra of **(A)** PEG-CHO, **(B)** DOX and **(C)** PEG-Schiff-DOX. The newly generated peak of Schiff base bond and the intricate peaks of DOX components fully testify the successful preparation of PEG-Schiff-DOX polymer.

The Schiff-linked PEGylate DOX prodrug was prepared following the synthetic route as shown in **Figure 1**. Previously, Sui et al. reported that DOX was conjugated into the PEG *via* an acid-cleavable hydrazone bond, but this doxorubicin prodrug system displayed the insensitive pH response, which exhibited a small DOX release with only 12% drug content within 72 h at pH 5.0. In this study, by means of the simple esterification reaction of 4-carboxybenzaldehyde and sequentially high-effective Schiff base reaction of DOX, the PEG-Schiff-DOX was feasibly obtained and confirmed by the ^1^H NMR spectra. **Figure 2A** clearly presented the assignments of PEG-CHO polymer. The signals at δ 8.0–8.3 ppm were attributed to the benzene components and the resonance peak of δ 10.2 ppm belonged to the proton of aldehyde group. Taking consideration of the intricate peaks for DOX, there was no clear peak attribution in **Figure 2B**. **Figure 2C** proved the successful conjugation of PEG with DOX, presenting the integrated assignments of PEG (δ 3.3 ppm and 3.5–4.1 ppm) and DOX moieties. The newly generated peak at δ 8.4 ppm for Schiff base bond and the completely disappeared signal of aldehyde group fully testified the successful preparation of PEG-Schiff-DOX polymer. Especially, the integration ratio of representative characteristic peaks further certified its exact and identical structure. The DOX content in the PEG-Schiff-DOX prodrug was calculated to be 38.1 wt%, higher than most of other reported DOX prodrugs, which was sufficient for therapeutic usage.

### Preparation, Stability and pH-Responsive Degradation of the Nanoparticles

**Figure 3:**
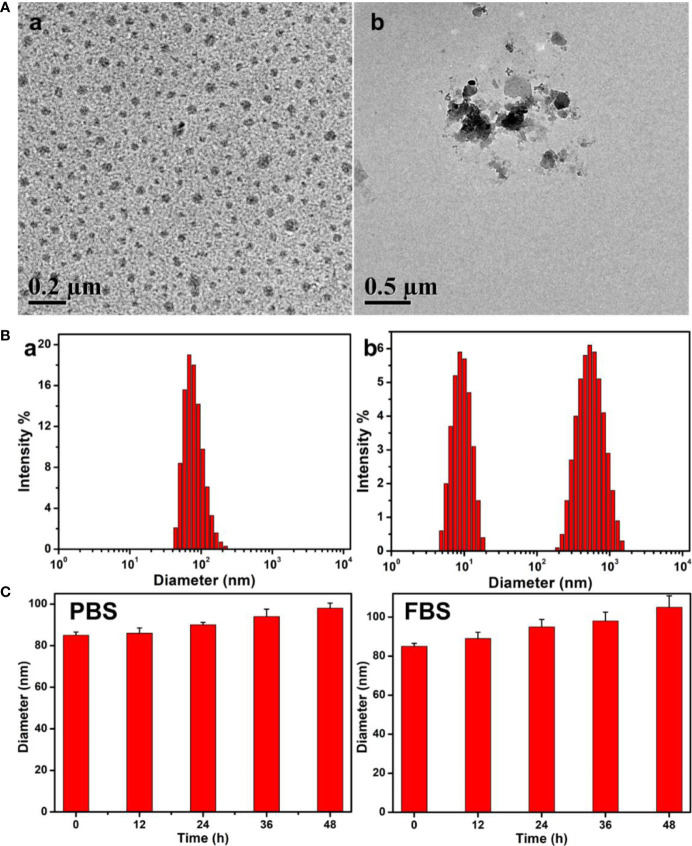
**(A)** TEM images and **(B)** Size changes of the PEG-Schiff-DOX nanoparticles before (a) and after (b) treatment of pH 5.0 PBS solutions for 4 h. **(C)** Size variations of the PEG-Schiff-DOX nanoparticles after incubation in PBS (pH = 7.4) and 20 wt% FBS solutions. These PEG-Schiff-DOX nanoparticles possessed pH-responsive drug release behavior within the tumor cells or tissues upon changes in physical and chemical environments, but they displayed good stability at physiological conditions for a long period.

The obtained PEG-Schiff-DOX could self-assemble into micellar nanoparticles in solutions because of its amphiphilic structure and high uniformity, which was well demonstrated by TEM image in **Figure 3A**-a. These nanoparticles had a z-average particle size of ca. 85 nm and a polydispersity index (PDI) of 0.22 by DLS result in **Figure 3B**-a. On account of the appropriate diameters (less than 200 nm) to keep lowered level of RES uptake, minimal renal excretion and efficient EPR effect for the passive tumor-targeting, PEG-Schiff-DOX prodrugs could be used as suitable drug carriers for tumor treatment.

These polymeric nanoparticles possessed outstanding storage stability that was important for the drug formulations. The acid-labile Schiff base linker between the hydrophobic DOX and the hydrophilic PEG made the PEG-Schiff-DOX nanoparticles susceptible to disassemble even under faintly acidic condition. The pH-responsive degradation behaviors of these nanoparticles at pH 5.0 solutions were monitored by DLS as summarized in **Figure 3B**-b. After incubated at pH 5.0 for 4 h, small molecules and aggregates were found, indicating the disassemble of polymeric nanoparticles. **Figure 3A**-b showed the degradation of micellar nanoparticles in the presence of pH 5.0 solutions, further confirming pH-induced cleavage of Schiff base bonds and dissociation of the aggregates. In addition, the diameter variations of PEG-Schiff-DOX micelles in PBS and FBS solutions were characterized using DLS, revealing their better storage stability in **Figure 3C**, which partly ascribed to the low CMC (0.018 mg/ml) of the prodrug. Besides, PEGylated prodrug nanoparticles themselves were negatively charged that could prevent aggregation by electrostatic repulsion and resistant protein absorption (8). Therefore, these kinds of pH-sensitive nanoparticles could keep stable at normal physiological conditions and degradable in response to acidic solutions, which may be used as the intelligent drug carriers in biomedical fields.

### *In Vitro* Drug Release of the Nanoparticles

**Figure 4:**
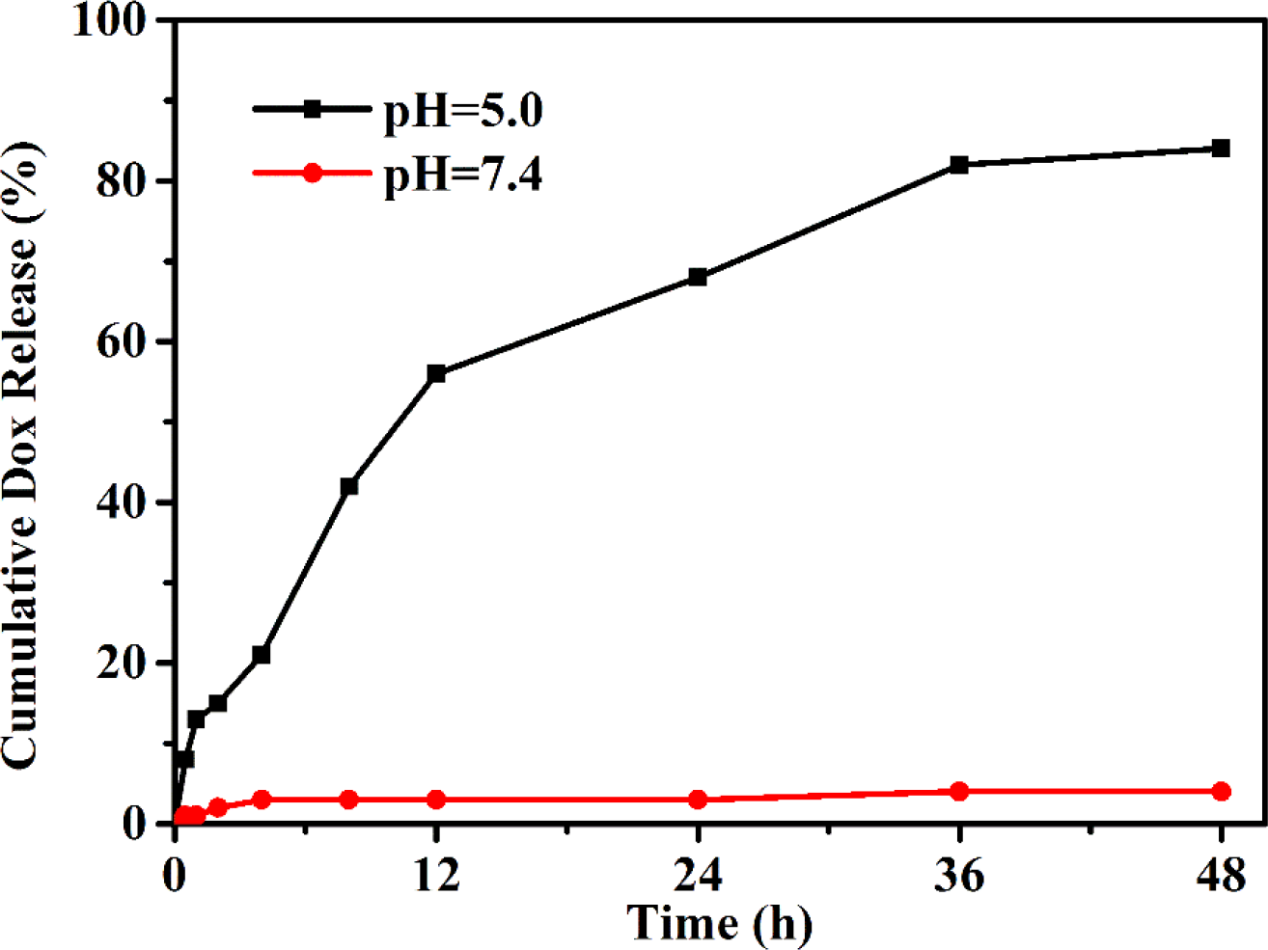
pH-triggered DOX release from PEG-Schiff-DOX nanoparticles at 37°C. The drug release profile reflects the pH responsivity of these nanoparticles that would maintain stable in neutral environment and quickly disassemble in acidic conditions.

The drug release profile of PEG–DOX nanoparticles was assessed under the physiological (PBS, pH 7.4) and acidic conditions (PBS, pH 5.0) to simulate the endo-lysosomal environment at 37°C. As depicted in **Figure 4**, a negligible DOX was released from the PEG-Schiff-DOX nanoparticles at pH 7.4 solutions while a much faster release of DOX at pH 5.0 solutions, thus achieving a high release content of 82.2%, which was attributed to the cleavage of Schiff base bonds to accelerate the DOX release. This pH change from extracellular environment (pH 7.4) to the endosomal compartments (pH 5.0) suggested that the PEG-Schiff-DOX nanoparticles would maintain stable and eliminate the premature burst release in blood circulation, while effectively promoting DOX release from its prodrug in the process of intracellular trafficking. In contrast to the conventional liposomal DOX formulations with poor stability and premature drug burst release, these pH-responsive nanoparticles exhibited the favorable stability, passive target capacity and tailored DOX release behavior could significantly promote the tumor endocytosis and enhance therapeutic effect.

### Cellular Uptake and Intracellular Localization of the Nanoparticles

**Figure 5:**
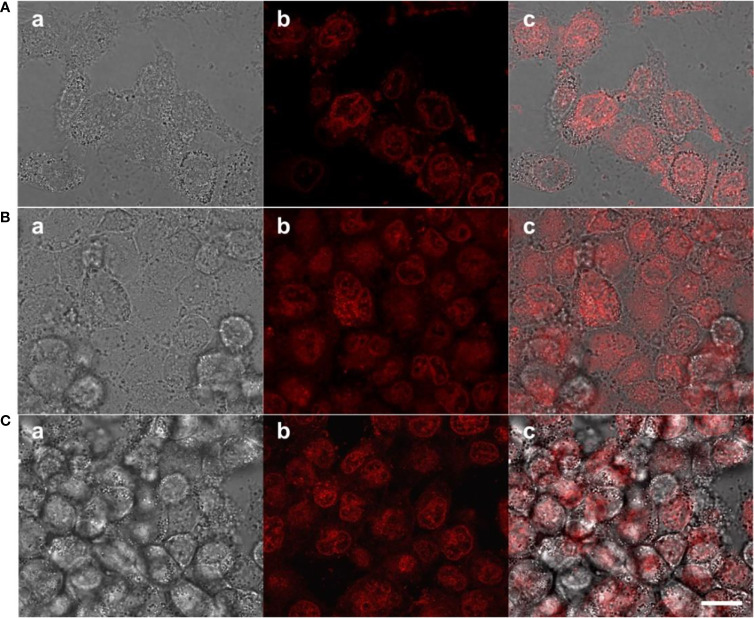
CLSM of MCF-7 cells after incubation with PEG-Schiff-DOX nanoparticles for **(A)** 1 h and **(B)** 4 h and free DOX drugs for **(C)** 1 h. a: bright field image, b: red fluorescence image and c: overlap of bright field and fluorescence images. Scale bars are 20 μm.

To determine the prodrugs internalized by tumors with therapeutic effects, cellular uptake process and intracellular drug release behavior were investigated using MCF-7 cells, because PEG-Schiff-DOX with inherent red fluorescence were directly observed with CLSM. As illustrated in **Figure 5A**, red fluorescence was apparently observed at the cytoplasm within the cells after incubation for 1 h, suggesting that these nanoparticles had been internalized easily internalized by the cells and concentrated in the endosome *via* the endocytosis. Along with the prolonged incubation time for 4 h, the intracellular fluorescence intensities tended to be stronger as observed in **Figure 5B**, suggesting the payload of the nanoparticles were gradually released into the nuclei. These findings indicated that the PEG-Schiff-DOX nanoparticles were successfully internalized by MCF-7 cells with efficient release of DOX from the nanoparticles and their further escape from the endosome/lysosome to the nucleus, revealing the applicability and practicability of this smart drug delivery system.

### *In Vitro* Antitumor Activity of the Nanoparticles

**Figure 6:**
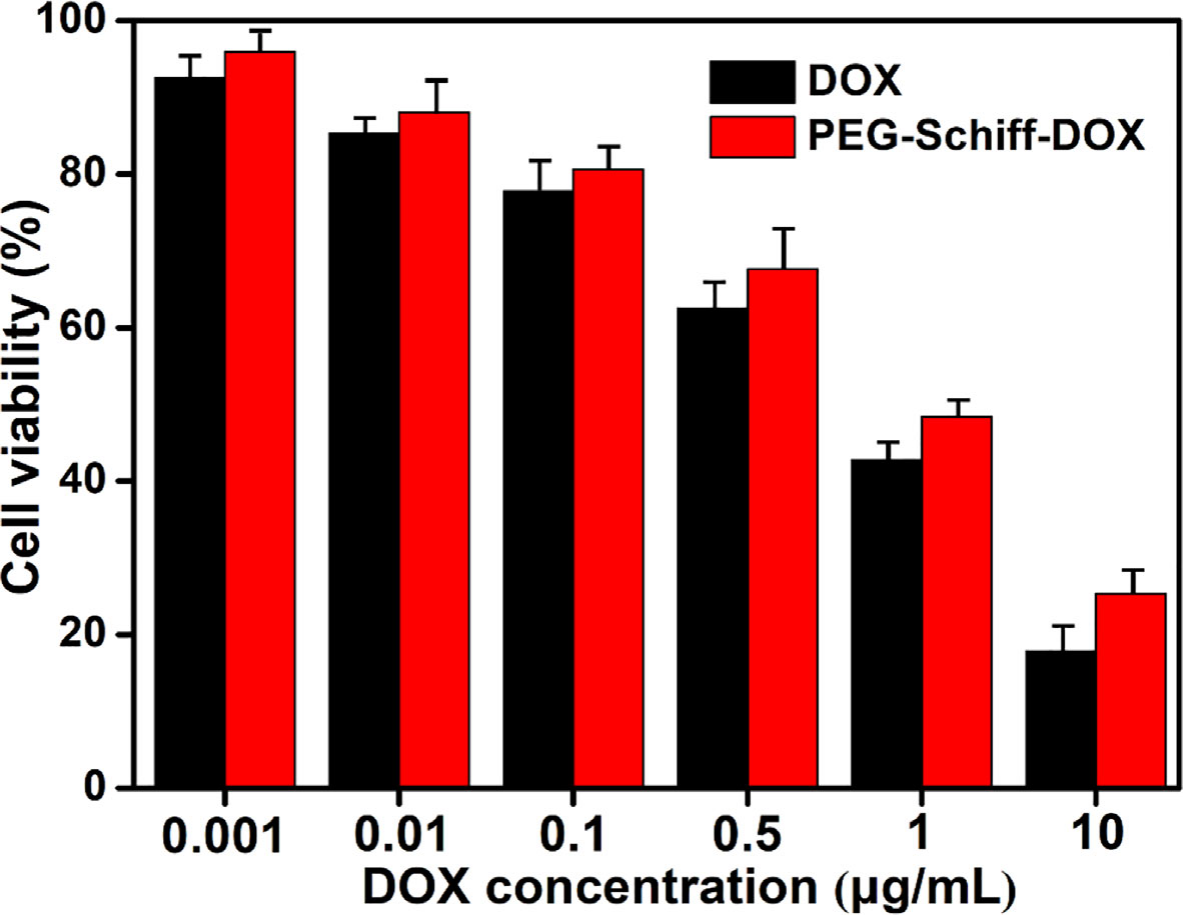
Cytotoxicity of MCF-7 cells following 24 h incubation with PEG-Schiff-DOX nanoparticles and free DOX molecules as a function of DOX dosages. All the data are presented as the average ± standard deviation.

The biocompatibility of the nanoparticles is an important issue in the fabrication of drug delivery system. Compared to the free DOX drug, PEG-Schiff-DOX nanoparticles could greatly enhance the drugs’ water solubility and stability, prolong their circulation in blood compartments, target cancerous tissues by passive accumulation *via* tumor’s EPR effect. Moreover, PEG is a well-known and widely used polymer carrier for drug delivery since it has been approved for clinical use. Therefore, PEGylated doxorubicin prodrugs have shown distinctly therapeutic advantages including better antitumor activity and fewer side effects than free drugs. *In vitro* cytotoxicity to MCF-7 cells of the nanoparticles was determined by CCK-8 assay. **Figure 6** showed high efficiency of antitumor activity toward MCF-7 cells after the incubation for 24 h. The viability of MCF-7 cells was relative to the DOX concentration. In a low concentration, PEG-Schiff-DOX nanoparticles had a similar toxicity as free DOX in a low concentration. While in a high concentration, DOX molecules could easily permeate the cellular and nuclear membranes by passive diffusion into cells, whereas PEG-Schiff-DOX nanoparticles were expected to be endocytosed to locate at the cells followed by endo-lysosomal escape and subsequent drug distribution in the cytosol and nucleus. In addition, **Figure 5C** showed that after incubation with free DOX at 1 h, the fluorescent intensity was obviously higher than that of PEG-Schiff-DOX nanoparticles incubated for 4 h, indicating the high concentration of free DOX internalized into the cells. In this case, the passive diffusion of free DOX molecules were much quicker than the above internalization processes of polymeric nanoparticles (34–38), demonstrating the relatively higher efficient antitumor capacity.

Although we have not performed the *in vivo* antitumor activity and cardiotoxicity, it is reasonable to conclude that the tumor growth can be effectively inhibited after DOX treatment. On account of the severe toxic side effects of free DOX drug, the body weight of animals can sharply decrease. In contrast, PEG-Schiff-DOX nanoparticles have significantly increased *in vivo* safety and exerted excellent therapeutic activity in animal models, which deserves further preclinical and clinical studies.

### Fabrication of DOX-Encapsulated PEG-Schiff-DOX Nanoparticles

Recent developments in nanomedicine for the cancer therapy have enabled programmable delivery of therapeutics by exploiting the stimuli-responsive properties of smart drug carriers. These therapeutic systems are designed with the relevant chemical and physical properties that respond to different triggers for enhanced anticancer efficacy, including the reduced development of drug-resistance, lower therapeutic dose, specific tumor target transport and spatiotemporally controlled release. Therefore, development of programmable nanocarriers for cancer therapy with particular emphasis on synergistic and sequential drug delivery systems is urgent (39, 40). Drug release kinetics are always associated with the intracellular drug concentration, drug action time and therapeutic effects. To actually simulate the tumor environment and maximum tumor growth inhibition, it is necessary to keep effective drug concentration for a time period as long as possible, especially at various requirement periods. Therefore, it is greatly desired to construct an ideal criterion that the designed drug delivery system should be able to reach high drug concentration to kill the tumor cells at an early period and exert an environmental responsive effect whenever needed over a long period. So, incorporation of encapsulated DOX and conjugated DOX in one drug delivery system would achieve a programmed drug release, which acquired the rapid release of encapsulated DOX to enhance intracellular drug concentration at first stage within a short time, and then responsive release of conjugated DOX to continue the effective treatment over a longer period, thereby resulting in the intelligent therapeutic effect.

**Figure 7:**
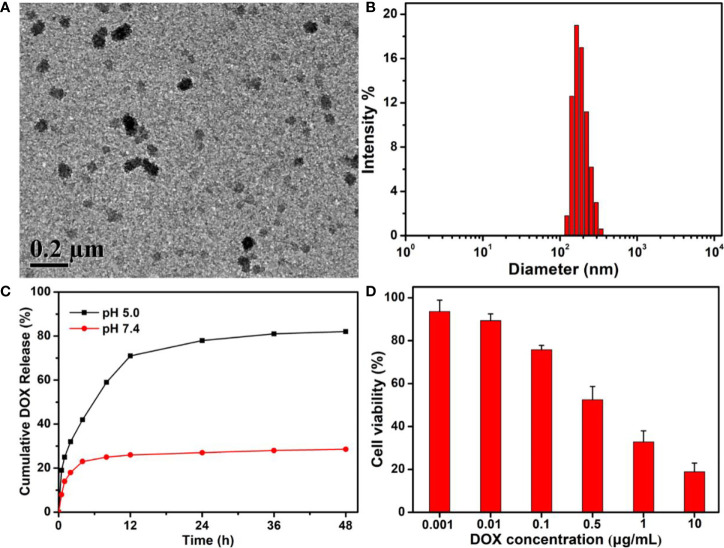
**(A)** TEM image, **(B)** DLS result and **(C)** *In vitro* drug release profile of DOX-encapsulated PEG-Schiff-DOX aggregates. **(D)** Cytotoxicity of MCF-7 cells following 24 h incubation with DOX-encapsulated PEG-Schiff-DOX aggregates as a function of DOX dosages. All the data are presented as the average ± standard deviation. These DOX-encapsulated PEG-Schiff-DOX nanoparticles possessed suitable structures and capacities with high drug loading, sufficient drug release and enhanced therapeutic effects of antitumor activity toward MCF-7 cells.

Using the traditional self-assembly strategy, free DOX drugs could be easily encapsulated into the PEG-Schiff-DOX nanoparticles due to the amphiphilic structures in solutions. The morphology, size and drug release behavior were clearly seen in **Figures 7A–C**. On account of the micellar structures, suitable hydrodynamic sizes, high drug loading and reasonable drug release capacity, these DOX-based nanoparticles with integrating physical encapsulation and chemical linkage can exhibit a two-phase programmed drug release behavior, namely, that the encapsulated DOX released rapidly at an early period to achieve high drug concentration to kill the tumors, and the conjugated DOX provided a responsive release requirement and longer release period to prolong the treatment time. However, an effective and practical carrier had not been required for the excessive drug content, because the instability of nanoparticles may lead to the undesirable drug release and induce the potential side effects. In fact, compared to the free DOX and PEG-Schiff-DOX nanoparticles, these DOX-encapsulated PEG-Schiff-DOX aggregates possessed more superior capacities with high drug loading, sufficient drug release and enhanced therapeutic effects of antitumor activity toward MCF-7 cells after the incubation of 24 h (**Figure 7D**), which can fully satisfy the clinical requirements with reducing drug leakage in the neutral environment of blood circulation and fast drug release in the acidic environment of tumor tissues and intracellular endosomal/lysosomal compartments.

## Conclusions

In summary, we developed a self-assembling PEGylated doxorubicin prodrug of PEG-Schiff-DOX *via* the conjugating hydrophobic DOX into a short PEG chain. This prodrug possessed good storage stability, high drug loading capacity and controlled release behavior. Incorporation of Schiff base into the polymeric architectures furnished the PEG-Schiff-DOX nanoparticles with pH responsiveness that maintained the structural integrity at neutral conditions and quickly disassembled in acidic conditions. Cytotoxicity assay indicated a fast internalization and a high cellular proliferation inhibition to MCF-7 cells, demonstrating excellent antitumor activities. In addition, this kind of nanoparticle could also encapsulate small free DOX drugs with high drug loading, sufficient drug release and enhanced therapeutic effects of antitumor activity toward MCF-7 cells, which may provide a promising option for developing translational DOX formulations and construction of multifunctional drug delivery systems in future.

## Data Availability Statement

The original contributions presented in the study are included in the article/supplementary material. Further inquiries can be directed to the corresponding authors.

## Author Contributions

JS, BX, and HY contributed equally to this reviewed paper. XW and ZY initiated the project. JS, BX, XL, YT, HY, and BW searched the data base, wrote, and finalized the manuscript. XW and ZY made important suggestions and helped in revising the paper. All authors reviewed and commented on the entire manuscript. All authors contributed to the article and approved the submitted version.

## Funding

This work was supported by National Natural Science Foundation of China (grant no. 51973226), Shenzhen Science and Technology Innovation Committee (grant no. JCYJ20180228163446770), and Youth Innovation Promotion Association CAS (grant no. 2019031).

## Conflict of Interest

The authors declare that the research was conducted in the absence of any commercial or financial relationships that could be construed as a potential conflict of interest.
